# Little evidence for an epidemic of myopia in Australian primary school children over the last 30 years

**DOI:** 10.1186/1471-2415-5-1

**Published:** 2005-02-11

**Authors:** Barbara M Junghans, Sheila G Crewther

**Affiliations:** 1 School of Optometry and Vision Science, University of New South Wales, Sydney, UNSW Sydney 2052. Australia; 2 School of Psychological Science, La Trobe University, Bundoora 3083, Australia

## Abstract

**Background:**

Recently reported prevalences of myopia in primary school children vary greatly in different regions of the world. This study aimed to estimate the prevalence of refractive errors in an unselected urban population of young primary school children in eastern Sydney, Australia, between 1998 and 2004, for comparison with our previously published data gathered using the same protocols and other Australian studies over the last 30 years.

**Methods:**

Right eye refractive data from non-cycloplegic retinoscopy was analysed for 1,936 children aged 4 to 12 years who underwent a full eye examination whilst on a vision science excursion to the Vision Education Centre Clinic at the University of New South Wales. Myopia was defined as spherical equivalents equal to or less than -0.50 D, and hyperopia as spherical equivalents greater than +0.50 D.

**Results:**

The mean spherical equivalent decreased significantly (p < 0.0001) with age from +0.73 ± 0.1D (SE) at age 4 to +0.21 ± 0.11D at age 12 years. The proportion of children across all ages with myopia of -0.50D or more was 8.4%, ranging from 2.3% of 4 year olds to 14.7% of 12 year olds. Hyperopia greater than +0.50D was present in 38.4%. A 3-way ANOVA for cohort, age and gender of both the current and our previous data showed a significant main effect for age (p < 0.0001) but not for cohort (p = 0.134) or gender (p = 0.61).

**Conclusions:**

Comparison of our new data with our early 1990s data and that from studies of over 8,000 Australian non-clinical rural and urban children in the 1970's and 1980's provided no evidence for the rapidly increasing prevalence of myopia described elsewhere in the world. In fact, the prevalence of myopia in Australian children continues to be significantly lower than that reported in Asia and North America despite changing demographics. This raises the issue of whether these results are a reflection of Australia's stable educational system and lifestyle over the last 30 years.

## Background

The prevalence of myopia is currently receiving worldwide attention as many recent studies report dramatic increases over the last 20 years [1,2]. Myopia and its aetiology is an interesting example of the intertwining of 'nature and nurture' with both genetics and life-style environment as important issues [3]. There is strong evidence indicating that genetic inheritance is a major contributor, both from the examination of prevalences across different racial backgrounds [4], from family pedigrees [5] and from twin studies [6]. However, there is increasing evidence suggesting that high heritability does not preclude rapid environmentally-induced increases in prevalence [7], rather, inherited factors are likely to both drive the susceptibility and resistance to environmentally-induced myopia [6,8].

Despite much research interest over the last half century, there have been surprisingly few well-designed epidemiological studies of refractive error with large numbers of randomly selected younger school children to form the basis of valid world wide comparisons of the earliest stages of development of myopia [3,9,10]. However, a group sponsored by the World Health Organisation in 2001 has devised a protocol to be used during studies of refractive error across different cultural and ethnic settings: the 'Refractive Error Study in Children' (RESC) [11].

In general, estimates of the prevalence of myopia have shown less increase in the Western world than in Asia, and less increase in rural than in urban populations [1,10,12-16]. Five very large studies across two decades and involving over 10,000 children in Taiwan are very important for understanding the changing prevalence of myopia in young Asian children (1.8% in 1986 rising to 12% in 1995 for 6 year olds, 40% rising to 56% for 12 year olds) [2]. A similar change is also reflected in Singaporean studies of myopia in military conscripts aged 17 years (26% to 83% from the late 1970s to the late 1990s as reviewed by [1]), of whom notably 82% were Chinese [17].

It has often been suggested that myopia is more prevalent in ethnic Chinese (reviewed [18]), but only relatively recent studies compare the prevalence of myopia in young ethnic Chinese children living either in China and in other countries [1,12,15,18-22]. For younger Chinese children aged around 5–7 years, the prevalence of myopia was found to range from under 5% in rural China [14,23] to 24% in Chinese Malays [20] and 30% in urban Hong Kong [19,22]. For older Chinese children aged 11–12 years, the prevalence ranged from 23% of rural Chinese[14,23] to 40% in urban China[12], 47% of Chinese Malays [20] and 57% in urban Hong Kong [22]. Japan has a similarly high prevalence of myopia in young school children estimated in recent times to be 43.5% of 12 year olds [24].

By comparison, the epidemiology of refractive error for young Australian school children is relatively well documented and presents a very different profile. A number of studies were carried out in the early 1970s and the 1980s on relatively large groups of unselected primary school children from the socio-economic extremes (generally aged 5 to 12 years), and indicated a prevalence of myopia ranging from approximately 3% to 13% (see Table 1) [25-29]. Two of those early studies investigated children largely from underprivileged, rural, families [26,27], and the other was of children from several, middle to upper socio-economic class private schools [25]. One smaller study was carried out in the mid 1980s on children from a representative selection of government schools in Brisbane [29], and would therefore have investigated children from a broader range of backgrounds. Interestingly, this latter study was the only Australian study to have determined refractive error under cycloplegia, yet yielded the highest prevalence of myopia. Thus, it has been difficult to determine whether the prevalence of myopia has increased in young school children in Australia as reported elsewhere. The majority of Australian residents are of Caucasian extraction living a very western lifestyle, leading one to expect the prevalence of myopia to be similar to that found in US or Europe. Yet, studies suggest that the prevalence of myopia in Australian primary school children is low by world standards [10].

In 2003 we reported the relative proportions of refractive errors in a large unselected primary school population of 2,535 children drawn from a very broad range of socio-economic backgrounds in Sydney, the largest city in Australia, in the early 1990s [30]. The children attended fourteen primary schools and two preschools. As in the earlier studies, the proportion of children with myopia greater than -0.50 DS spherical equivalence, as determined by non-cycloplegic retinoscopy, was found to be low by world standards (1.0% of 4 year olds rising to 8.3% of 12 year olds). We have now analysed the prevalence of refractive error in a new similar group of 1,936 children unselected primary school children drawn generally from the same area as our first study.

## Methods

The study design is a retrospective examination of records of the Vision Education Centre (VEC) [31] school vision screenings (so named because parents were not present to ratify history) conducted in the Clinic of the School of Optometry and Vision Science, UNSW. Approvals for the study and permission to approach schools were obtained from the Committee for Use of Humans in Research at the University of New South Wales (UNSW), Sydney, Australia. The protocols adhered to the tenets of the Declaration of Helsinki. Parents or guardians were provided with an information sheet and requested an outline of known symptoms. Signed consent was required prior to a child's participation.

### Sampling and recruitment

Permission was obtained from the NSW Department of Education and the NSW Catholic Education Office to approach all schools in the eastern region of Sydney (some thirty coeducational primary schools) to send entire classes to the VEC. A flyer was sent describing the VEC science excursion and age-appropriate eye examination, inviting Years 1, 3 and 5 particularly to participate.

The group of 1,936 children examined came from the eastern suburbs along the southern beaches of Sydney, and may be thought of as randomly selected with little likelihood of bias to the data as individual classes were free to respond. Children were drawn from twelve government and non-government primary schools and one pre-school and attended the clinic only once. During the 1996 Australian Bureau of Statistics census 14,785 children aged 4 to 12 years were recorded in this region (Randwick and Waverley precincts of Eastern Sydney) who came from a very broad range of ethnic and socio-economic backgrounds present, where 37 different languages might be spoken in the home [32]. This was reflected in the children attending VEC. Census data indicate approximately 9% of the children in the current study were likely to be of Asian origin [32], a figure supported by our interpretation of family name for each child [30]. Participation in the eye examinations was typically well over 90% for each class, with teachers reporting non-participation to be predominantly due to illness on the day. Less than 3% of parents intentionally prevented participation, even if eye care had previously been sought. This particularly high participation rate was largely due to the attraction of a an age-appropriate student-centred hands-on science lesson about eyes and vision [31] delivered alongside the eye examination.

### Clinical examination

The comprehensive optometric examination by experienced paediatric practitioners included all age-appropriate tests meeting Australian Optometric Competency Standards, except that parents/guardians were not present to ratify history. Refractive error was determined by non-cycloplegic retinoscopy with optical fogging while the child maintained fixation on a distant non-accommodative (6 metre) target. In most cases refractive status was confirmed by subjective refraction. Other tests included letter visual acuity at 6 m and 33 cm, cover test for strabismus, motilities, saccades, pupil reactions, near point of convergence, heterophoria, stereopsis, accommodative facility, colour vision and ophthalmoscopy.

### Justification of choice of testing procedures

Cycloplegic retinoscopy was not undertaken for many reasons including the fact that VEC studies started prior to the 2000 convention suggesting use of cycloplegic retinoscopy for studies of refractive error prevalence [11]. Secondly, the VEC visit was meant as an excursion and the children had to return to normal classes with near work demands after the morning outing. Thirdly, it was important for comparison purposes to use refractive data procured under the same conditions as that used for the earlier groups of children. Fourthly, an initial evaluation without cycloplegia is necessary in order to understand daily function. Fifthly, non-cycloplegic retinoscopy was only one component of the exam. Outcomes regarding a decision to refer would not alter for most children had a cycloplegic refraction been carried out, as several other near function tests that would also indicate the possible existence of latent hyperopia or pseudo-myopia were included. Lastly, the degree of refractive error may in fact be influenced by cycloplegia (see Discussion for elaboration [33-38]).

Autorefractors were not employed as hand-held versions were unavailable when the first cohort was seen. Equally as important, there is no convincing evidence that the proportion of myopes identified in the sample would have changed [39].

### Comparison with earlier data

To compare the estimated prevalence of myopia in this urban population of 'Australian children' over the last decade, this more recent 2000s data set was analysed against data from an earlier cohort of 2,322 children with similar demographics seen in the early 1990's, using the same testing protocols and seen at the same venue [30]. The optometric results of that earlier cohort have previously been reported [40], and it was noted that 7.1% of those children were already wearing spectacles [30], indicating that our recruitment procedure did not preclude children already under the care elsewhere. The data for any child examined in both cohorts was deleted from the earlier data set to avoid bias in the analysis. The mean date of assessment for this last 2000s cohort was September 2000, and for the early 1990s cohort was June 1992. Thus, the average gap between assessments of children from the two cohorts was 8 years and 3 months.

### Statistical analyses

Data was analysed by Analysis of Variance ANOVA (StatView software). Only refractive data from right eyes was used for the current refractive class analysis, as the correlation between right and left eye refractions was extremely high (p < 0.0005). The preferred criterion to define myopia in this study is that used clinically in Australia: a spherical equivalent equal to or more minus than -0.50 D. However, as myopia more minus than -0.50 D has occasionally been used to define myopia in epidemiological studies [13,19,41], analyses using the criterion 'myopia more minus than -0.50 D' were also performed for comparison. Hyperopia was defined as spherical equivalents greater than +0.50 D. Thus, emmetropia for this study was defined as refractions in the range -0.25 to +0.50 dioptres spherical equivalence inclusive. Means are quoted with the associated standard error.

## Results

The records of 1,936 children aged 4 to 12 years from a non-clinical unselected population examined during the six years from March 1998 to May 2004 were analysed retrospectively to estimate the prevalence of different types of refractive error. Primary schools of their own choice sent more children from years 1, 3, and 5, which resulted in unequal numbers of children in each of the age groups. There were 925 boys and 951 girls, and the relative numbers for both males and females in each age group are shown in Table 2. For 59 children, the gender was not indicated on the record card and could not be inferred with certainty from the given name. The data not associated with gender has only been included in analyses entitled 'All' as shown in Tables 2 and 3. Mean age was 8.36 years. The relative proportions of the different classifications of refractive error for all children combined (including those of unknown gender) for each age group are shown in Table 2.

**Figure 1:**
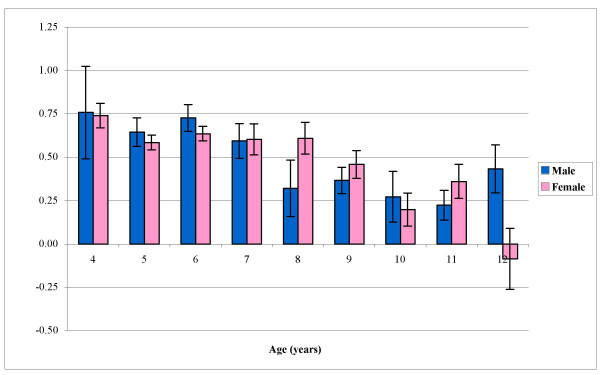
Histogram of mean right eye spherical equivalent refractive error by age and gender ('2000s' data). Error bars represent standard error.

The mean spherical equivalent refraction of all 1,936 children was +0.45 ± 0.02 DS, however it should be noted that there is a preponderance of children aged 5–6, 9 and 11 years old corresponding with Years 1, 3, and 5 of primary school. Overall, there was no significant difference in spherical equivalent refractive error between girls and boys (p = 0.697). In general, mean refraction demonstrates a highly significant shift towards less hyperopia with increasing age (p < 0.0001) from 0.73 ± 0.1DS for 4 year olds to 0.21 ± 0.11 for 12 year olds, however this is more noticeable after the age of 9 years as seen in Fig. 1. With increasing age, more children are found in the emmetropic category and fewer in the low hypermetropic category.

**Figure 2:**
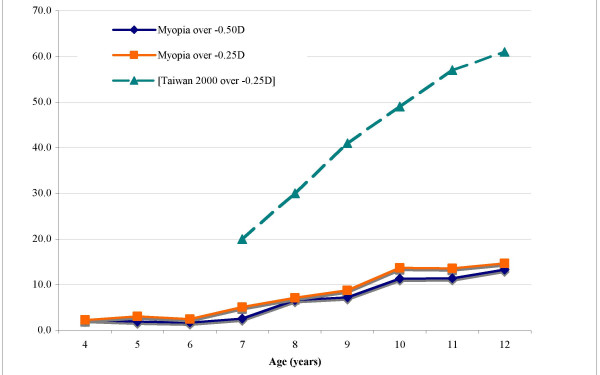
Line graphs of the percentage Sydney children with myopia by age using the alternate criteria of more minus than -0.50 D spherical equivalence (blue line) or more minus than -0.25DS spherical equivalence (orange line), and, the percentage of Taiwanese children seen in the year 2000 more minus than -0.25D (aqua dashed line) taken from Lin et al, Ann Acad Med 2004 33:27–33.

A summary of the relative proportions of myopia and hyperopia for this cohort of children of all ages seen during the six years ('2000s' data) is given in Table 3. The majority of children screened are emmetropic by our criteria: 53.0% averaged across all ages. The proportion of children manifesting moderate to high degrees of hypermetropia (≥+1.50 DS) is 6.2% across all ages. Only 6.9% of children of all ages had refractive errors more minus than -0.50 DS, ranging from 2.3% of 4 year olds to 13.3% of 12 year olds (Fig. 2). If the more liberal definition of myopia is applied (myopia equal to or more minus than -0.50), then 8.4% of all children were myopic (ranging from 2.3% of 2 year olds to 14.7% of 12 year olds). Only 0.8% of the 1,936 children were more than -4.00 DS myopic.

### Comparison with previous data

**Figure 3:**
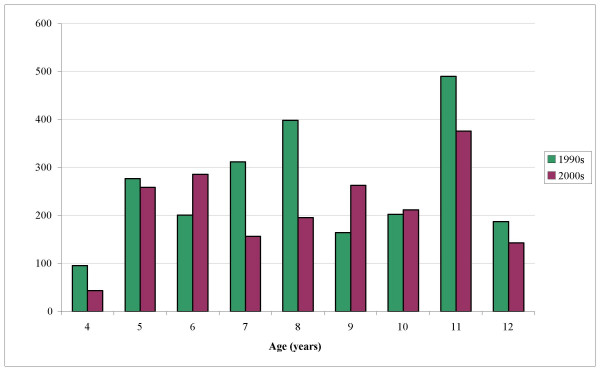
Histogram of the number of children in each age group for data from the current study (2000s) and the previously reported early 1990s data [30].

**Figure 4:**
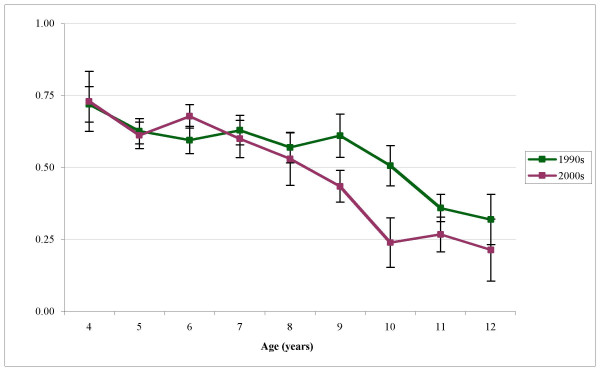
The mean right eye spherical equivalent refractive error by age for data from the current study and the previously reported early 1990s data [30]. Error bars represent standard error.

The number of children per age group for the two cohorts is shown in Fig. 3, and notably, the age profile differs slightly between cohorts, though the mean age of 8.37 years for the earlier cohort was similar to that of this later cohort. The mean and standard error for right eye spherical equivalent refractive error by age is shown for each cohort in Fig. 4.

A three-way analysis of variance was carried out for cohort, age and gender. There was a main effect for age (p < 0.0001), but no main effect for either cohort (p = 0.134) or gender (p = 0.61). However, there were age/gender/cohort interactions that indicate a trend towards an increasing shift away from the hyperopic refraction in the later cohort.

## Discussion

An analysis of the prevalence of refractive errors in young school children in eastern Sydney during the last thirteen years has been presented. The latest data gathered from 1,936 unselected primary school-aged children in the last 6 years, indicates that the prevalence of myopia remains quite low compared to that reported for the western world and Asia, especially as refractive error was established by non-cycloplegic retinoscopy (as will be discussed later). These findings are not significantly different (p = 0.13) to our previous report [30] indicating that 6.5% of 2,535 unselected children aged 4 to 12 years seen in the early 1990s were myopic by at least 0.50 D. Notably, those children were of similar socio-economic and ethnic status drawn from the same region of Sydney and seen at the same Centre using the same testing protocol.

Therefore, if we take the total 4,258 children seen since 1990, the relative frequency of refractive error across all is: 54.2% emmetropic by our criteria, 32.3% low to moderate hyperopes, 5.3% myopic greater than -0.50D spherical equivalence and 7.4% myopic by at least -0.50 DS. The number with myopia of at least -4.00 DS was an extremely small 0.6%.

### The prevalence of myopia in Sydney primary school children compared to the rest of the world

As alluded to in the introduction, the proportion of Sydney children with myopia is dramatically less than in Asia. Indeed, the proportion appears significantly lower than in the USA [41] and Canada [42] (4% and 6% of 6 year olds respectively, or 20% of 12 year olds in USA), but higher than urban India with only 4.4% of all school children under 16 years myopic [13] and higher particularly than in other less developed countries [10].

In the past, a lack of internationally accepted definitions for 'myopia' has hampered valid comparisons across the various studies [10]. Commonly the criteria 'greater than -0.50 DS' or 'at least -0.50 DS' are employed. However, our separate analyses using both of these criteria only resulted in a difference of 1.5% of all children included as myopic, in keeping with other dual analyses [13,41], and is low either way when compared with Asia or North America.

Comparison across studies is also difficult when only an 'overall' mean refraction is presented covering all children in a study, due to the well known increasing prevalence of myopia with age. Indeed, the comparison of data from our own two data sets is confounded to some extent by the slightly different age profiles for each cohort. However, in neither cohort was the age range nor mean significantly different, so the similar proportion of myopes is not unexpected.

### Comparison of refractive error with and without a cycloplegic agent

The question of optimal ocular conditions for comparison of the prevalence of refractive errors remains controversial. A cycloplegic agent is typically proposed as the gold standard [3,43,44] in the belief that it will eliminate ciliary muscle action or spasm, and thus unmask latent hyperopia or pseudomyopia. Thus, the use of a cycloplegic would be firstly predicted to lead to a decrease in the prevalence of myopia, and an increase in the prevalence of hyperopia. However, as a cycloplegic also leads to associated mydriasis and the introduction of unpredictable spherical aberrations, it is arguable that cycloplegia will induce unpredictable errors. In fact, Gao et al [38] in 2002 reported significant changes in the refractive components of children's eyes under conditions of deep cycloplegia and mydriasis that were greatest in hyperopic eyes and smallest in myopic eyes, adding no definitive evidence as to the relative efficacy of cycloplegia.

Thus there appears to be no scientific concurrence regarding the efficacy of cycloplegia for studies on the prevalence of myopia [35-37], with several major studies electing to use cycloplegia (see review in [10,9,11]) and others not [18-21,23,42,45]. Presumably this design variability exists because there is no decisive evidence indicating a difference between refractions determined with and without a cycloplegic agent in eyes that have a myopic refraction. In general, a more positive retinoscopic finding is reported under cycloplegia, though considerable individual variation is seen including a myopic shift in some [33,35-37,46]. Not surprisingly, the differences noted decreased both with age and with less positive refraction.

As our refractive data was derived from non-cycloplegic retinoscopy we readily concede that mean refractive error may be less hyperopic than if a cycloplegic had been used. However, we suggest that as the influence of a cycloplegic is uncertain and is of least concern for myopes, the estimated prevalence of myopia will not be significantly altered by our decision to not use a cycloplegic. In support of this notion are new conference data from Rose et al [47,48] reporting refractive status ascertained by cycloplegic autorefraction in over 1,000 children aged 6–7 years from across the same city of Sydney. They reported values of 'around 3%' for the prevalence of myopia of at least 0.50D [47], and then the value of 1.5% for myopia of 'approximately 0.50D' [48] with a participation rate between 73 and 80%. From Table 2 it can be seen that 2.4% of our 6 year olds in the current study were at least 0.50D myopic – a value that is strikingly similar.

### Demographics versus lifestyle

Worldwide patterns of the prevalence of myopia suggest significant differences are likely to be due to the different demographics and lifestyles [1,10,49]. Zadnik [41] concedes that the increase in numbers of myopic children in the US Orinda study may be due to changing ethnic demographics. The apparent slight increase in myopia in Australia reported in the current study may also be in part accounted for by our changing ethnic demographics in urban areas. However demographics and ethnic compositions are unlikely to be responsible for the large changes reported in Asian and some other western countries [1,50].

Whatever way it is argued, our results indicate little evidence for an epidemic of myopia although there is a developmental trend towards an earlier decrease in hyperopia to the point of myopia. Thus, the question of whether it is a matter of lifestyle, or perhaps familial environmental stress, or more, remains. Certainly, the education system and housing has changed little in Australia the last 30 years. By comparison, most Asian children participating in myopia epidemiological studies reportedly are more likely to live in high-rise residential blocks [17] and have strong demands at school to memorize along with parental and peer pressure to do well, and for some, a competitive entrance examination to enter school [19,51].

## Conclusions

It is concluded that despite some differences in methodology across earlier studies, the prevalence of myopia in young Australian school children does not appear to have increased significantly over the last 30 years if one allows for the change in ethnic demographics. It is also proposed that an explanation for the large increase in prevalence of myopia reported in other countries must include questions relating to lifestyle in addition to genetic propensity.

## Competing interests

The author(s) declare that they have no competing interests.

## Authors' contributions

SC conceived and designed the study, jointly analysed the results and worked the drafts through to the final version. BJ coordinated the study, collated the clinic records, jointly analysed the results, researched the background for the paper, prepared the draft manuscript and was responsible for presentation. Both authors read and approved the manuscript.

## Pre-publication history

The pre-publication history for this paper can be accessed here:



## Figures and Tables

**Table 1:** Prevalence of myopia in Australia school children.

Authors	Year	N	Place	Ages	Method	Criterion	Prevalence %
Robbins & Bailey [25]	1975	1,243	Private schools	4, 8, 12	Non-cycloplegic retinoscopy	Myopia≥-0.50D "suspected of having myopia"	5.6% 4 yrs 6.4% 12 yrs
Amigo, McCarthy & Pye [26]	1976	1,166	Rural underprivileged	7 to 12	Non-cycloplegic retinoscopy	"any tendency for myopia"	3.4%
Walters [27]	1981 1982 1983	2,055 1,187 1,722	Rural underprivileged	4 to 14	Non-cycloplegic retinoscopy	Myopia≥-0.50D	4.40% 3.03% 2.67%
Macfarlane [29]	1987	877	Representative government schools	6 to 11	Cycloplegic retinoscopy	Not stated	13%
Junghans et al [30]	Early 1990s	2,535	14 government & church schools	4 to 12	Non-cycloplegic retinoscopy	Myopia≥-0.50D	2.0% 4 yrs 10.9% 12 yrs Mean 6.5%

**Table 2:** Estimated prevalence of refractive error (%) by age in children aged 4 to 12 years.

**Age**	**Myopia**			**Emmetropia**	**Hyperopia**		**N**		
	**More than -3.75**	**-0.50 to -3.75**	**of -0.50**	**-0.25 to +0.50**	**+0.75 to +1.25**	**More than +1.25**	**Female**	**Male**	**All**

4	0.0	2.3	0.0	43.2	50.0	4.5	24	16	44
5	0.4	1.5	1.2	49.8	41.3	5.8	121	129	259
6	0.0	1.7	0.7	47.6	43.4	6.6	145	133	286
7	0.0	2.5	2.5	54.8	33.1	7.0	72	80	157
8	1.0	5.6	0.5	49.0	29.6	14.3	100	85	196
9	0.8	6.5	1.5	56.3	29.7	5.3	133	128	263
10	2.4	9.0	2.4	54.7	27.8	3.8	149	88	212
11	0.5	10.9	2.1	58.0	24.5	4.0	146	189	376
12	2.1	11.2	1.4	57.3	22.4	5.6	61	77	143

**Table 3:** Mean estimated prevalence of myopia and hyperopia in children aged 4 to 12 years.

	**Female**		**Male**		**All**	
	
**Refractive Category:**	N	%	N	%	N	%
**Myopia ≤-4**	8	0.8	8	0.9	15	0.8
**Myopia -3.75≤x≤-0.75**	58	6.1	58	6.3	118	6.1
**Myopia of -0.50**	11	1.2	17	1.8	29	1.5
**Emmetropia -0.25≤x≤0.50**	504	53.0	501	54.2	1030	53.2
**Hyperopia +0.75≤x≤+1.25**	320	33.6	282	30.5	624	32.2
**Hyperopia ≥+1.50**	50	5.3	59	6.4	120	6.2
Total	951	100	925	100	1936	100
